# Patient Care Assistants Making Meaning of Their Work in Nursing Homes

**DOI:** 10.1093/geroni/igaf122.4169

**Published:** 2025-12-31

**Authors:** Justina Knowles

**Affiliations:** University of The Bahamas, Nassau, New Providence, Bahamas, The

## Abstract

The purpose of this study was to describe the meaning that patient care assistants (PCAs) ascribe to their work in nursing homes. This study was needed to close existing knowledge gaps, such as the existence of obsolete findings that cannot guide modern practice, lack of generalizability of the outcomes of previous studies, and inadequate empirical evidence to support policies and programs. Guided by the meaning of work level model, the purpose of this qualitative case study was to evaluate what meanings PCAs attach to their work in a profession that is undervalued and underpaid. Data were collected from in-depth interviews with 10 PCAs and four RNs to understand how the benefits of meaning making help PCAs adopt better work culture and institutional practices, and what characteristics RNs perceive as nurturing a positive meaning-making experience. Thematic analysis revealed that a positive workplace culture, strong organizational practices, effective collaboration, caring relationships, opportunities for professional development, and resilience and adaptation contributed to the meaning-making experience for PCAs. The perspectives of PCAs and RNs regarding their work may facilitate the creation of protocols, policies, and programs for empowering PCAs to create positive meaning of their work in nursing homes. The findings may also promote improved care outcomes among older adults in nursing homes. Positive social change may occur when health care teams understand the significant role and value of care that PCAs deliver in nursing homes.

